# Editorial: Tendencies and new horizons for digital health use in the gynecological cancer patient journey

**DOI:** 10.3389/fonc.2024.1411664

**Published:** 2024-04-23

**Authors:** Mohamed Otify

**Affiliations:** Department of Gynaecological Oncology, Liverpool Women's Hospital, Liverpool, United Kingdom

**Keywords:** predictive modeling, radiomics, online health information-seeking behavior, gynaecologic oncology, machine learning (ML), personalized medicine

The landscape of gynecologic oncology is constantly shifting, with new diagnostic methods, treatment options, and prognostic tools emerging to improve patient care. In this editorial, we spotlight two recent studies that offer valuable insights into gynecologic malignancies and showcase the potential of innovative approaches to enhance patient outcomes (1–3).

Xiong et al. delve into the online health information-seeking habits of gynecologic oncology patients in China, particularly on widely used platforms such as Douyin and Baidu. Their findings reveal that a blend of demographic and psychological factors can foretell the probability of these patients turning to the Internet for information. Age, marital status, and anxiety levels emerged as noteworthy predictors, with a combined model exhibiting strong predictive performance (AUC=0.841). This study emphasizes the significance of grasping patients’ information-seeking behaviours in our digital era and suggests that personalized communication strategies could bolster patient education and engagement.

Meanwhile, Yang et al. explore the potential of MRI-based radiomics models for preoperative risk classification of endometrial endometrioid adenocarcinoma. They construct a radiomics nomogram that weaves traditional radiomics features, deep learning image features, and clinical data. The nomogram displayed impressive predictive performance in the training set (AUC=0.923) and validation set (AUC=0.842), surpassing models relying solely on traditional radiomics or deep learning features. This study underscores the promise of radiomics and deep learning techniques in providing non-invasive, preoperative risk stratification, thereby facilitating individualized treatment planning.

Both studies contribute to the burgeoning corpus of research to elevate patient care in gynecologic oncology (1–3). The insights gleaned from predictive modelling and radiomics have the potential to revolutionize clinical practice, empowering healthcare providers better to understand their patients’ needs and tailor management strategies accordingly. As the field advances, validating these findings further and exploring their implementation in real-world settings is imperative.

Future research should broaden the scope of predictive models by incorporating additional factors that influence online health information-seeking behaviour, such as health literacy and trust in online resources. Furthermore, integrating radiomics and deep learning techniques into clinical decision-making processes warrants further investigation, with an emphasis on prospective validation and the development of user-friendly tools for clinicians.

In conclusion, the studies by Xiong et al. and Yang et al. illuminate the potential of innovative approaches to propel gynecologic oncology research and enhance patient care. By harnessing the power of predictive modelling and radiomics, we can better understand our patients’ needs, provide personalized care, and ultimately improve outcomes for those facing gynecologic malignancies. As we navigate the ever-evolving landscape of gynecologic oncology, we must remain open to novel ideas and continue to push the boundaries of what is possible in patient care (1–3).

## Author contributions

MO: Writing – original draft, Writing – review & editing.
